# The Burden of Diabetes-Related Preventable Hospitalization: 11-Year Trend and Associated Factors in a Region of Southern Italy

**DOI:** 10.3390/healthcare9080997

**Published:** 2021-08-04

**Authors:** Giuseppe Di Martino, Pamela Di Giovanni, Fabrizio Cedrone, Michela D’Addezio, Francesca Meo, Piera Scampoli, Ferdinando Romano, Tommaso Staniscia

**Affiliations:** 1 Department of Medicine and Ageing Sciences, “G. d’Annunzio” University of Chieti-Pescara, 66100 Chieti, Italy; tommaso.staniscia@unich.it; 2 Unit of Hygiene, Epidemiology and Public Health, Local Health Authority of Pescara, 65100 Pescara, Italy; 3 Department of Pharmacy, “G. d’Annunzio” University of Chieti-Pescara, 66100 Chieti, Italy; pamela.digiovanni@unich.it; 4 School of Hygiene and Preventive Medicine, “G. d’Annunzio” University of Chieti-Pescara, 66100 Chieti, Italy; cedronefab@gmail.com (F.C.); michela.daddezio@gmail.com (M.D.); Francesca.meo10@gmail.com (F.M.); piera.scampoli@gmail.com (P.S.); 5 Department of Infectious Diseases and Public Health, “La Sapienza” University of Rome, 00185 Roma, Italy; ferdinando.romano@uniroma1.it

**Keywords:** epidemiology, diabetes, hospitalization, complications, PQI, prevention

## Abstract

(1) Introduction: Diabetes care is complex and delivered by different care providers in different settings across the healthcare system. Better coordination through all levels of care can lead to better outcomes and fewer hospitalizations. Prevention quality indicators (PQIs) for diabetes allow us to monitor diabetes-related avoidable admissions. The aim of this research is to assess the trend of diabetes-related preventable hospitalizations and associated risk factors in a southern Italian region. (2) Methods: The study considered all hospital admissions performed from 2008 to 2018 in the Abruzzo region, Southern Italy. Data were collected from hospital discharge records. Four different indicators were evaluated as follows: short-term complications (PQI-01), long-term complications (PQI-03), uncontrolled diabetes (PQI-14) and lower-extremity amputations (PQI-16). Joinpoint models were used to evaluate the time trends of standardized rates and the average annual percent change (AAPC). (3) Results: During study period, 8660 DRPH were performed: 1298 among PQI-01, 3217 among PQI-03, 1975 among PQI-14 and 2170 among PQI-16. During the study period, PQI-01and PQI-04 showed decreasing trends. An increasing trend was showed by PQI-16. (4) Conclusions: During an 11-year period, admissions for short-term diabetes complications and for uncontrolled diabetes significantly decreased. The use of standardized tools as PQIs can help the evaluation of healthcare providers in developing preventive strategy.

## 1. Introduction

Diabetes imposes a substantial burden on society in terms of higher medical costs, lost productivity, premature mortality and intangible cost in the form of reduced quality of life [1]. The global prevalence of diabetes among adults over 18 years of age has risen from 4.7% in 1980 to 9.3% in 2019 [2,3], and annual diabetes-related healthcare costs are at least USD 147 billion worldwide [3]. Diabetes care is complex and delivered by different care providers in different settings across the healthcare system. Better coordination through all levels of care is hypothesized to result in better health outcomes and fewer hospitalizations [4]. In most countries, the great part of diabetes care is provided in primary care. Primary care is supposed to provide care close to patients with no access barriers, comprehensive to the needs of patients, coordinate care through all healthcare levels and is continuous over time [5]. Reducing potentially preventable hospitalizations is important for increasing the quality of care and containing hospital costs. In fact, patients who have a continuous relationship with their care providers have overall better health outcomes in terms of fewer emergency department visits and better control of chronic diseases [6]. In particular, some studies showed that patients that frequently accessed a primary care provider have less of a chance of being hospitalized for diabetes complications [7,8]. For these reasons, diabetes-related hospital admissions are frequently used as a quality indicator for health services and primary care settings. To rank the value of healthcare services, the Agency for Healthcare Research and Quality (AHRQ) created four prevention quality indicators (PQIs) for diabetes, which allowed for monitoring through the hospital discharge records (HDRs) of diabetes-related avoidable admissions [9]. In addition, the knowledge of factors associated with diabetes-related preventable hospitalization (DRPH) can be help in improving preventive strategies and in achieving a better quality of care. The aim of this research is to assess the trend of diabetes-related preventable hospitalizations and associated risk factors in a southern Italian region.

## 2. Materials and Methods

### 2.1. Study Design

The study considered all hospital admissions performed from 2008 to 2018 in the Abruzzo region, Southern Italy. Abruzzo has over 1.2 million inhabitants and has 29 hospitals, with 4 tertiary hospitals. Data were collected from all hospital discharge records (HDR), which include information on the patient’s demographic characteristics, a diagnosis-related group code (DRG) used to classify the admission and a maximum of 6 diagnoses (1 principal diagnosis and up to 5 secondary diagnosis) and 6 procedures (1 principal procedure and up to 5 secondary procedures) followed during the hospitalization and coded as per the International Classification of Disease, 9th Revision, Clinical Modification (ICD-9-CM). The following socio-demographic variables were also collected: age, citizenship, marital status, scholarship, hospitalization length, mode of admission, mode of discharge and costs. To compute the preventable admission rate for diabetes, AHRQ definitions [9] were followed, including four different indicators: short-term complications (PQI-01), long-term complications (PQI-03), uncontrolled diabetes (PQI-14) and lower-extremity amputations among patients with diabetes (PQI-16). Because the definition of PQI-16 can overlap with that of PQI-01 and PQI-03, all preventable hospitalizations that met the criteria for PQI-16 and other hospitalization types (either PQI-01 or PQI-03) were considered as PQI-16, in order to eliminate the risk of double counting. In order to evaluate risk factors associated with each PQI, all diseases belonging to the Charlson Comorbidity Index (CCI) were extracted according to the methodology proposed by Quan et al. [10,11]. The CCI assesses the comorbidity level by taking into account both the number and severity of pre-defined comorbid conditions. It provides a weighted score of a patient’s comorbidities that can be used to predict short-term and long-term outcomes such as function, hospital length of stay and mortality rates. The total score in the CCI was calculated by summing the assigned weights of all comorbid conditions presented by the patient.

### 2.2. Ethical Approval

The study was conducted in conformity with the regulations on data management of the Regional Health Authority of Abruzzo and with the Italian Law on privacy (Art. 20–21 DL 196/2003) published on the Official Journal n. 190 of 14 August 2004. Data were encrypted prior to the analysis at the regional statistical office, where each patient was assigned a unique identifier. This identifier eliminates the possibility to trace the patient’s identity. According to the Italian legislation, the use of administrative data does not require any patient written informed consent.

### 2.3. Statistical Analysis

Quantitative variables were summarized as mean and standard deviation (SD) or median and interquartile range (IQR) according to their distribution. Qualitative variables were summarized as frequency and percentage. The hospitalization rate was calculated by dividing the number of hospital admissions by the total number of the at-risk Abruzzo population in the first observed year, standardized for age and gender. The joinpoint model (Joinpoint version 4.6.0.0, 2018) was used to evaluate the time trends of standardized rates, the direction and the intensity of the (linear) trend and the average annual percent change (AAPC). AAPC is considered a summary measure of the trend over a given fixed time interval that is computed as a weighted average of the annual percent change emerging from the joinpoint model, using weights equating to the length of the period interval. The final model is based on linear segments connected at joinpoints that represent the best fit of observed data. In order to evaluate risk factors associated to DRPH, a logistic regression model was performed, using all comorbidities of CCI as covariates. For all analyses, a *p*-value ≤ 0.05 was assumed to indicate statistical significance (two-tailed). Statistical analyses were performed using STATA v14 software (StataCorp LLC, College Station, TX, USA).

## 3. Results

### 3.1. Characteristics of Admissions

During study period, 8660 DRPH were performed: 1298 among PQI-01, 3217 among PQI-03, 1975 among PQI-14 and 2170 among PQI-16. Gender was equally distributed between males and females across all PQI considered, as reported in Table 1. Patients admitted for PQI-16 were older compared to other PQIs. The majority of patients were discharged at home, but PQI-01 more frequently showed in-hospital mortality. The LOS was similar among all PQIs, and the median ranged from 6 for PQI-03 to 8 for PQI-16 and PQI-01.

### 3.2. Trend in PQIs Admissions

**Figure 1:**
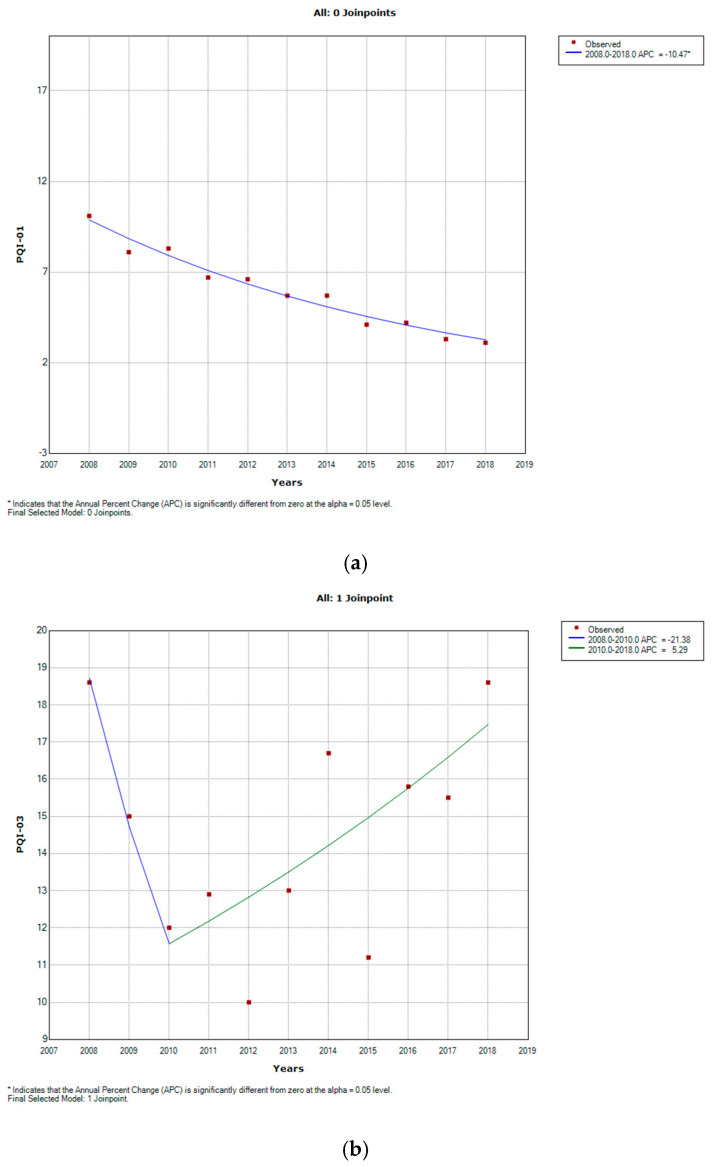

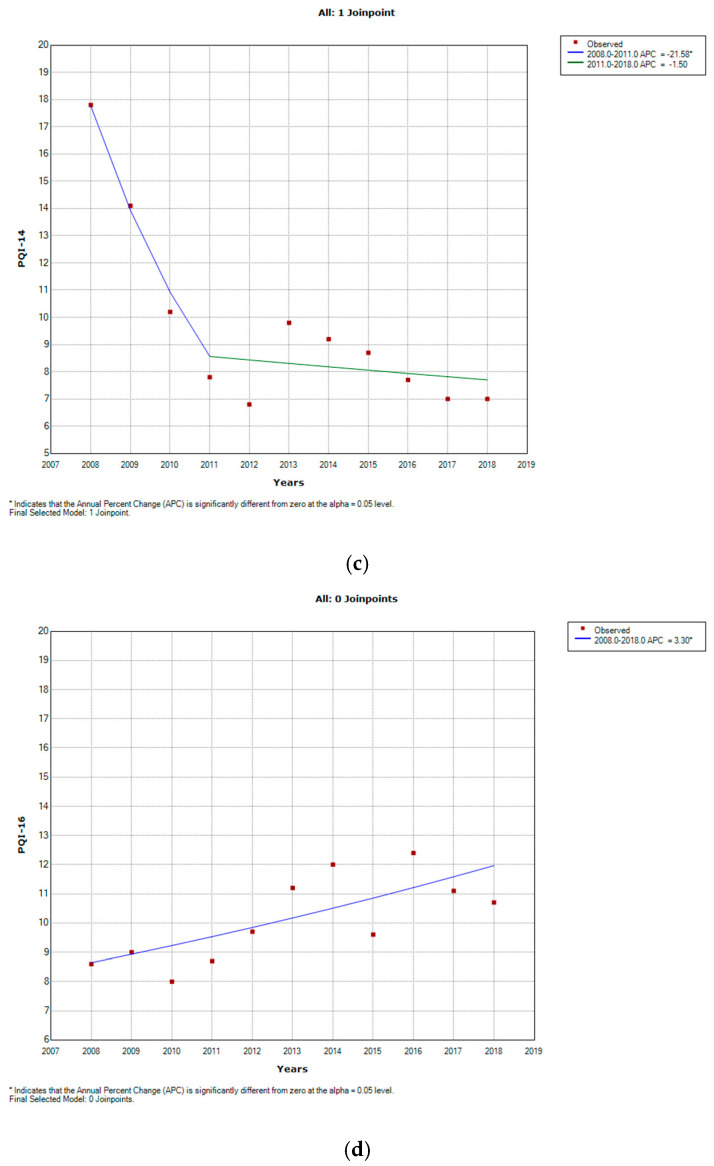
Joinpoint regression analyses reporting annual age and gender standardized rates and average annual percentage change (AAPC) observed for diabetes-related preventable hospitalization from 2008 to 2018. (**a**) PQI-01; (**b**) PQI-03; (**c**) PQI-14; (**d**) PQI-16; * Indicates that the APC is significantly different from zero at the alpha = 0.05 level.

During study period, as showed in Figure 1, PQI-01 showed a decreasing trend from 10.1 cases/100,000 in 2008 to 3.1 cases/100,000 in 2018 (AAPC −10.9, CI −12.3–9.5). A similar trend was shown by PQI-14, from 17.8 cases/100,000 to 7.0 cases/100,000 (AAPC −8.2, CI −14.2–2.8). An increasing trend was shown by PQ-16, from 8.6 to 10.7 cases/100,000 (AAPC 3.3, CI 0.9–5.8). A stable trend over the period was shown by PQI-03, from 18.6 to 18.6 cases/100,000 (AAPC −0.7, CI −8.3–7.8), with a significant decrease during first two years of observation (AAPC −21.4, CI −49.6–22.7) and a regular increase during the last period (AAPC 5.3, CI −0.4–11.3).

### 3.3. Factors Associated with PQIs

Considering factors associated with PQIs, male gender was significantly associated only with PQI-03. Among CCI comorbidities, patients with acute myocardial infarction were less likely to occur in DPRH, as shown in Table 2. Peripheral vascular disease was significantly associated with PQI-03 and PQI-16, with aOR = 15.51 (95% CI 14.25–16.88) and 5.05 (95% CI 4.50–5.67), respectively. Dementia showed an association with PQI-01 (aOR = 1.73, 95% CI 1.28-2.35) and PQI-14 (aOR = 1.40, 95% CI 1.06–1.85). Additionally, liver diseases were associated with PQI-01(aOR = 1.83, 95% CI 1.48–2.26) and PQI-14 (aOR = 3.35, 95% CI 2.93–3.84).

## 4. Discussion

This study evaluated the burden of DRPH, analyzing all hospital admissions performed in a southern Italian region during an 11-year period. DRPH heavily affected the hospital management, particularly in the region as Abruzzo, with a high prevalence of diabetes [12]. PQIs can be used as the measures to compare the healthcare quality of local-level providers within a European country characterized by universal coverage such as Italy [13], and they can reveal information about the community health system outside the hospital environment, indirectly measuring the quality of primary healthcare [14]. Lower-extremity amputations and long-term complications remained as the most frequent conditions of DRPH during the period of study, followed by short-term complications and lower-extremity amputations. All DRPH mainly affected patients aged 65 and over, particularly among patients aged over 75 years, in line with the previous literature [15]. The majority of patients were discharged at home, but PQI-01 and PQI-04 resulted in higher in-hospital mortality. These results are in line with the previous literature, which reports an in-hospital mortality rate of 5%–16% for short-term complications [16]. The in-hospital mortality rate for lower-extremity amputation was inferior compared to previous studies [17,18].

During study period, PQI-01 and PQI-14 showed a significant reduction, according with the recent literature [19,20]. It can be explained by improved out-hospital diabetes care, in terms of improved drug and lifestyle interventions and personalized care. About 15 diabetes care centers are still working in Abruzzo, shifting the diabetes management out of hospitals and also involving general practitioners. Having multidisciplinary and personalized care is known as an important factor in improving diabetes care and avoiding short-term complications [19].

Improving care of diabetes during study period did not result in changes in long-term diabetes-related complications. In fact, PQI-03 and PQI-16 did not show a reduction. Probably, management intervention requires more time to impact the incidence of long-term diabetes complications [20,21,22].

Among comorbidities, patients who underwent lower-extremity amputation were more frequently males and more frequently had a peripheral vascular disease (60.14%). These results confirmed that male gender was associated with lower-extremity amputation and that peripheral vascular disease was one of the strongest predictors of this complication, in line with the previous literature [20,21,22]. Dementia resulted to be associated with short-term diabetes complications and uncontrolled diabetes admission, probably due to the poor compliance of these patients in diabetes management [23], as is true for other diseases [24].

In addition, the coexistence of liver complications would accelerate the deterioration of patients with diabetes. Liver cirrhosis and diabetes influence each other. Thus, in addition to pharmacological treatments and lifestyle interventions, effective control of liver disease might assist in a better management of diabetes [25].

Microvascular diabetes complications were associated with both renal disease and peripheral vascular diseases. This relation can explain the significant association of renal disease and amputations, as also known in the recent literature [26,27].

These findings showed as several conditions and diseases are associated to PQIs, highlighting the important relation between primary care and diabetes care. In addition, a recent study highlighted the impact of hyperglycemic state at the admission on patients’ comorbidities and its influence on short-term outcomes. This point highlights the importance of primary care on diabetes management, not only in avoiding diabetes hospitalization, but also to improve patients outcomes during hospitalization [28].

This article added value on this topic in terms of knowledge about trends in the results of primary healthcare actions in a southern Italian region, concerning the unfavorable outcome (avoidable hospitalizations). It can contribute to identify diseases that need consistent actions of continuous quality improvement. This analysis can be in improved in future by analyzing the spatial analysis aiming to investigate socio-economic factors implied in PQIs. Furthermore, it may be the starting point for the analysis of other PQI rates such as the chronic obstructive pulmonary disease or heart failure [14].

### Strengths and Limitations

The strengths of this study were the large sample considered during an 11-year period, the use of a standardized data source such as HDR and the use of a useful and recognized tool such as PQI.

The results of this study should be considered in the light of some limitations. In this study, HDRs were used, thus considering only in-patient information. Several factors, such as duration of diabetes, drug therapy, quality of care and other clinical information, could not be accounted for. This lack of information can lead to unmeasured confounders hampering the multivariate analysis. Furthermore, an under-reporting of diabetes diagnosis is possible, causing an inaccurate estimate of hospitalization rates.

## 5. Conclusions

During 11-year period, admissions for short-term diabetes complications and for uncontrolled diabetes significantly decreased. These results highlight the importance of improving ambulatory care and diabetes management to reduce preventable hospitalization. Further studies with a longer follow-up period are needed to evaluate the improvement of long-term DRPH. Regulatory agencies and policymakers should endorse metrics and expectations for the hospital management of diabetes, as has been done for ambulatory diabetes management, to incentivize health systems to incorporate acute care into their diabetes population health programs, in particular for heavy complications such as diabetic foot and amputations. Knowing factors associated with DRPH can lead to the tailored management of diabetes primary care. The use of a standardized tool as PQIs can help the evaluation of healthcare providers in developing a preventive strategy and improving diseases management.

## Figures and Tables

**Table 1:** Characteristics of included admissions.

*N* (%)	PQI-01	PQI-03	PQI-14	PQI-16
Total discharge	1298	3217	1975	2170
Male	594 (45.76)	1525 (47.40)	955 (48.35)	1092 (50.32)
Female	704 (54.24)	1692 (52.60)	1020 (51.65)	1078 (49.68)
Age category				
*18–34*	158 (12.17)	350 (10.88)	227 (11.49)	23 (10.69)
*35–44*	123 (9.48)	337 (10.48)	204 (10.33)	187 (8.62)
*45–54*	166 (12.79)	385 (11.97)	234 (11.85)	239 (11.01)
*55–64*	170 (13.10)	469 (14.58)	280 (14.18)	350 (16.13)
*65–74*	255 (19.65)	668 (20.76)	405 (20.51)	468 (21.57)
*≥* *75*	426 (32.82)	1008 (31.33)	625 (31.65)	694 (31.98)
Length of stay, median (IQR)	8 (4–17)	6 (3–11)	7 (4–11)	8 (4–15)
Discharge type				
*At home*	1089 (83.90)	2957 (91.92)	1778 (90.03)	1857 (85.58)
*Long-term care facilities*	55 (4.24)	37 (1.15)	19 (0.96)	91 (4.19)
*Other*	24 (1.85)	38 (1.18)	25 (1.27)	61 (2.81)
*Death*	130 (10.02)	185 (5.75)	153 (7.75)	161 (7.42)

**Table 2:** Multivariable logistic regression models to evaluate DRPH-associated factors.

Variables	PQI-01	PQI-03	PQI-14	PQI-16
	aOR (95% CI)	aOR (95% CI)	aOR (95% CI)	aOR (95% CI)
**Male vs. Female**	0.93 (0.84–1.04)	**0.97 (0.40–0.83)**	1.03 (0.94–1.13)	1.05 (0.96–1.15)
**Age category**				
18–34	0.91 (0.73–1.13)	0.86 (0.74–1.00)	0.95 (0.79–1.15)	0.90 (0.74–1.08)
35–44	0.82 (0.65–1.04)	0.96 (0.82–1.12)	0.98 (0.81–1.19)	0.84 (0.69–1.02)
45–54	REF.	REF.	REF.	REF.
55–64	**0.72 (0.58–0.89)**	**0.85 (0.74–0.98)**	0.84 (0.70–1.00)	0.98 (0.83–1.16)
65–74	**0.75 (0.62–0.92)**	**0.83 (0.73–0.95)**	0.85 (0.72–1.00)	0.93 (0.79–1.09)
>75	**0.77 (0.64–0.92)**	**0.79 (0.69–0.89)**	**0.82 (0.70–0.96)**	0.86 (0.73–1.00)
**Charlson Comorbidities**				
IMA	**0.16 (0.07–0.34)**	**0.24 (0.17–0.33)**	**0.27 (0.16–0.43)**	**0.47 (0.34–0.65)**
CHF	**0.44 (0.33–0.58)**	**0.24 (0.19–0.30)**	**0.28 (0.21–0.37)**	**0.26 (0.20–0.33)**
Peripheral Vascular	0.64 (0.43–0.93)	15.51 (14.25–16.88)	0.76 (0.57–1.02)	5.05 (4.50–5.67)
Cerebro-vascular	0.85 (0.70–1.02)	0.46 (0.39–0.53)	**0.76 (0.65–0.90)**	**0.42 (0.34–0.51)**
Dementia	1.73 (1.2 –2.35)	0.91 (0.68–1.20)	1.40 (1.06–1.85)	0.52 (0.33–0.82)
COPD	**0.35 (0.26–0.46)**	**0.28 (0.22–0.34)**	**0.49 (0.40–0.60)**	**0.56 (0.47–0.68)**
Reumatic diseases	0.64 (0.26–1.55)	0.61 (0.33–1.13)	1.56 (0.97–2.50)	0.71 (0.33–1.52)
Peptic Ulcer	0.88 (0.33–2.38)	0.66 (0.32–1.37)	0.40 (0.13–1.27)	0.16 (0.02–1.20)
Hemi-paraplegia	0.94 (0.35–2.54)	**0.20 (0.05–0.83)**	0.31 (0.07–1.25)	0.19 (0.02–1.40)
Malignacy	0.72 (0.50–1.03)	**0.37 (0.26–0.53)**	0.83 (0.63–1.10)	0.37 (0.23–0.59)
Liver disease	1.83 (1.48–2.26)	0.91 (0.75–1.10)	**3.35 (2.93–3.84)**	0.28 (0.18–0.44)
Metastatic	0.63 (0.37–1.09)	**0.02 (0.00–0.16)**	**0.40 (0.23–0.68)**	**0.10 (0.02–0.42)**
AIDS/HIV	-	2.19 (0.66–7.26)	0.80 (0.11–5.90)	-
Renal disease	0.87 (0.71–1.08)	**0.83 (0.73–0.96)**	**0.80 (0.67–0.96)**	**1.52 (1.35–1.71)**

Significant OR were bolded. All models were adjusted for gender, age and CCI.

## Data Availability

Note applicable.
